# Association about dietary vitamin C intake on the risk of ovarian cancer: a meta-analysis

**DOI:** 10.1042/BSR20192385

**Published:** 2020-08-07

**Authors:** Yuhang Long, Hui Fei, Sumei Xu, Jianzhen Wen, Lihua Ye, Zhaojuan Su

**Affiliations:** 1 Department of Gynecology and Obstetrics, The Fifth Affiliated Hospital of Sun Yat-Sen University, Zhuhai, Guangdong 519000, China; 2 Department of Gynecology and Obstetrics, The Seventh Affiliated Hospital of Sun Yat-Sen University, Shenzhen, Guangdong 518000, China; 3 Medical Record Department, The Fifth Affiliated Hospital of Sun Yat-Sen University, Zhuhai, Guangdong 519000, China

**Keywords:** Dietary, Meta-analysis, Ovarian cancer, Vitamin C

## Abstract

Changes in dietary vitamin C intake have been related to the risks of various cancers. However, the association between dietary vitamin C intake and the risk of ovarian cancer has not been fully determined. A meta-analysis was performed to evaluate the relationship between vitamin C intake and ovarian cancer risk. Observational studies that evaluated the association between vitamin C intake and ovarian cancer risk were identified via systematic search of PubMed and Embase databases. A random-effect model was used to combine relative risk (RR) with corresponding 95% confidence intervals (CIs). As a result, 16 studies (5 cohort studies and 11 case–control studies) with 4553 cases and 439,741 participants were included. Pooled results showed that dietary vitamin C intake had non-significant association on the risk of ovarian cancer (RR = 0.95, 95%CI = 0.81–1.11, *I*^2^ = 52.1%, *P*_for heterogeneity_ = 0.008). Subgroup analyses according to characteristics including geographic location and study design showed consistent results with the overall result. In summary, findings from the present study indicated that dietary vitamin C intake is not associated with the risk of ovarian cancer.

## Introduction

According to Globocan’s estimate in 2018, cancer is the second leading cause of death worldwide, with an estimated 9.6 million deaths [1]. Ovarian cancer is still the most deadly gynecologic malignancy [2]. Meanwhile, it is also the leading cause of cancer-related death in women [2,3]. Previous paper estimated that there were 22,440 new cases and 14,080 deaths of ovarian cancer in 2017 [2]. Therefore, primary prevention of ovarian cancer is necessary. Although ovarian cancer is confirmed to be associated with many genetic factors [4,5], some dietary factors may also affect the development the risk of ovarian cancer. Dietary vitamin C intake has been linked to many cancers, such as pancreatic cancer [6,7], cervical neoplasia [8], renal cell carcinoma [9], esophageal cancer [10], prostate cancer [11], and so on. However, no comprehensive meta-analysis was performed to explore the relationship about vitamin C intake on the risk of ovarian cancer recently. Up to now, several studies have investigated the effectiveness of dietary vitamin C intake on the risk of ovarian cancer, and these results should be re-evaluated to provide robust pooled results. Therefore, the current meta-analysis of available observational studies was conducted to determine the role of vitamin C intake on the risk of ovarian cancer.

## Materials and methods

### Data sources, search strategy, and selection criteria

**Figure 1:**
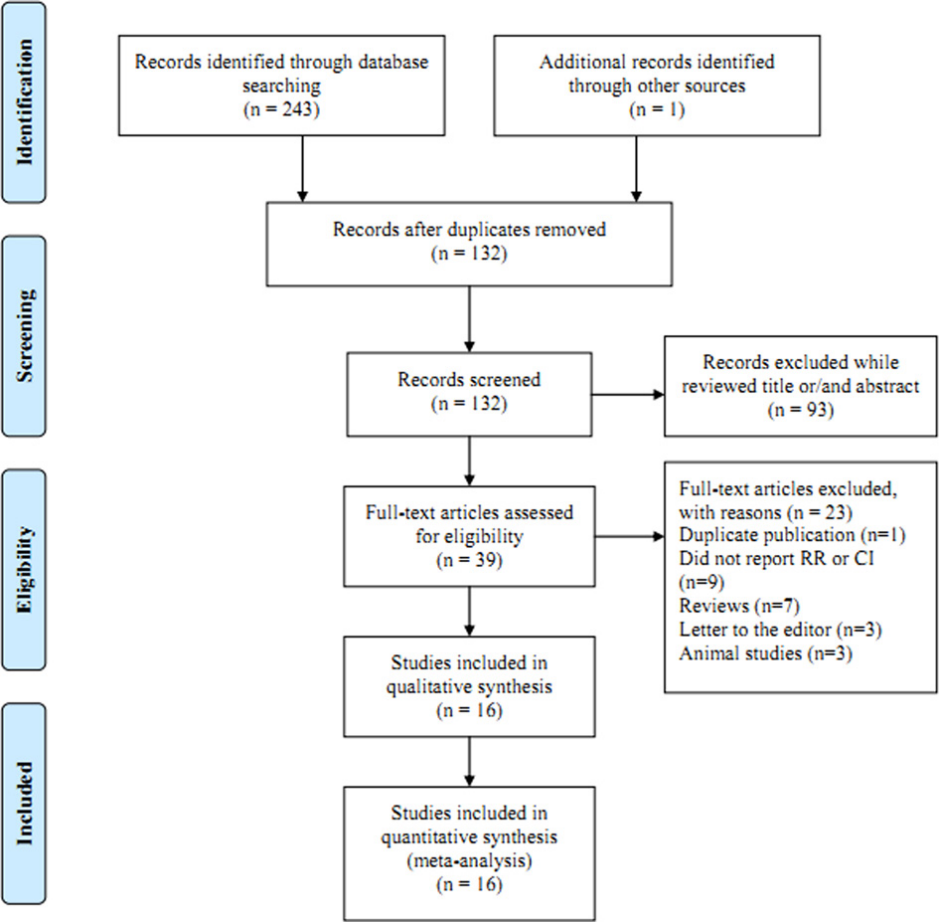
The flow diagram of screened, excluded, and analyzed publications

The present study was performed and reported according to the Preferred Reporting Items for Systematic Reviews and Meta-Analysis Statement issued in 2009 [12]. Electronic searches for relevant studies about vitamin C intake and the risk of ovarian cancer were conducted of PubMed and Embase from their inception to May 31, 2019. The search terms included ‘vitamin C’ OR ‘vitamin*’ combined with ‘ovarian cancer’ OR ‘ovarian tumor’. We manually searched the reference lists of the retrieved studies to identify any other eligible papers (Figure 1).

Two authors independently conducted the literature search and selected the studies by reading the titles, abstracts, and full-text articles, and any disagreement was resolved by an additional author until consensus was reached.

Studies were included if they met the following criteria: (1) Patients: patients diagnosed with ovarian cancer and ≥18 years of age; (2) Study design: all the observational studies were acceptable; (3) Interested and outcomes: the studies should assess the association about dietary vitamin C intake on the risk of ovarian cancer; (4) Data: the study should provide the available data of relative risk (RR) and 95% confidence intervals (CI). Furthermore, we only included studies that explore the relationship about dietary vitamin C intake only, but not vitamin C supplement, on the risk of ovarian cancer.

The exclusion criteria were as follows: (1) case reports, conference abstracts, letters, editorials, reviews; (2) overlapping or duplicate studies; (3) irrelevant studies; (4) no available data of RR and 95%CI.

### Data collection

One author conducted the data collection according to a standard flowchart, while another author checked it. If any disagreement was detected, they discussed the issue until consensus was reached. The data collected included the family name of the first author, publication year, country, cases and participants, age, category of vitamin C intake, the value of RR and 95%CI, adjustment for factors.

### Statistical analysis

The combined RR and 95%CI was pooled using STATA software (version 10.0, College Station, TX, U.S.A.) with a random-effects model [13]. Heterogeneity among the included studies was calculated using *I*-square and *P* values for *Q* statistic, and significant heterogeneity was defined as an *I*-square >50.0% or *P* < 0.10 [14,15]. The robustness of the pooled results was measured using a sensitivity analysis by sequential exclusion of individual trials. Funnel plot [16] and Egger test [17] were used to evaluate potential publication bias. The inspection levels for all pooled results were 0.05.

## Results

### Literature search

The initial electronic searches produced 243 articles and one article was identified from the reference of reviews; of them, 205 were excluded due to irrelevant topics and duplication. The remaining 39 full articles were reviewed; of them, 16 articles [18–32] involving 4553 cases and 437,689 participants were included in the final analysis. Fourteen of the included studies come from North America, one from Europe and one from Asia. Five of the 16 articles were cohort design and the remaining 11 articles were case–control design. Table 1 summarizes the general characteristics of the patients and studies.

**Table 1:** Characteristics of the included studies about vitamin C intake on ovarian cancer risk

Study, year	Design	Age	Participants, Cases	Country	Category	RR (95%CI)	Adjustment
Slattery et al., 1989	PBCC	20–79	577,85	United States	>159.1 vs. <97.8 mg/d	0.7 (0.3–1.4)	Adjusted for age, body mass index of weight/height^2^, and number of pregnancies. All dietary variables are in separate logistic models.
Tzonou et al., 1993	HBCC	18–75	389,189	Greece	Highest vs. lowest	0.90 (0.76–1.06)	Adjusted for age, years of schooling, parity, age at first birth, menopausal status as well as for energy intake
Kushi et al., 1999	Cohort	55–69	29,083,139	United States	>321.9 vs. <129.2 mg/d	1.05 (0.63–1.76)	Adjusted for age, total energy intake, number of live births, age at menopause, family history of ovarian cancer in a first-degree relative, hysterectomy/unilateral oophorectomy status, waist-to-hip ratio, level of physical activity, cigarette smoking (number of pack-years), and educational level
Cramer et al., 2001	PBCC	>50	1,065,549	United States	>337 vs. ≤97 mg/d	1.00 (0.66–1.53)	Adjusted for total caloric intake, age, site, parity, body mass index, oral contraceptive use, family history of breast, ovarian or prostate cancer in a first-degree relative, tubal ligation, education, and marital status
Fairfield et al., 2001	Cohort	30–55	80,326,301	United States	Q5 vs. Q1	1.22 (0.83–1.81)	Adjusted for age, body mass index (kg/m^2^), duration of oral contraception use, smoking history, parity, history of tubal ligation, and caffeine intake
Fleischauer et al., 2001	HBCC	≥18	419,168	United States	>180 vs. <100 mg/d	1.04 (0.57–1.92)	Adjusted for age, parity, body mass index, total caloric intake, and family history of breast and/or ovarian cancer
McCann et al., 2001	HBCC	20–87	1,921,496	United States	>250 vs. ≤112 mg/d	0.69 (0.47–1.03)	Adjusted for age, education, region of residence, regularity of menstruation, family history of ovarian cancer, parity, age at menarche, oral contraceptive use, and total energy intake
Salazar-Martinez et al., 2002	HBCC	20–79	713,84	Mexico	≥184 vs. ≤78 mg/d	1.28 (0.72–2.28)	Adjusted for age, total energy intake, number of live births, recent changes in weight, physical activity, and diabetes
McCann et al., 2003	PBCC	40–85	820,124	United States	>244 vs. <123 mg/d	0.82 (0.42–1.59)	Adjusted for age, education, total months menstruating, difficulty becoming pregnant, oral contraceptive use (ever/never), menopausal status, and total energy
Zhang et al., 2004	HBCC	18–75	906,254	China	≥140.25 vs. ≤66.50 mg/d	0.31 (0.18–0.53)	Adjusted for terms for age, locality, education, family income, BMI, total energy intake, tobacco smoking, alcohol consumption, ovarian cancer in first degree relatives, parity, menopausal status, and oral contraceptive use
Tung et al., 2005	PBCC	45–75	1,165,558	United States	Q4 vs. Q1	0.89 (0.62–1.26)	Adjusted for age, ethnicity, study site, education, oral contraceptive pill use, pregnancy status, tubal ligation, and energy intake by polytomous logistic regression (histologic type), or unconditional logistic regression (all other variables)
Silvera et al., 2006	Cohort	40–59	89,835,264	Canada	>206 vs. <115 mg/d	0.90 (0.58–1.37)	Adjusted for age, menopausal status, use of oral contraceptives, body mass index, education, participation in vigorous physical activity, energy intake at baseline, study center, and randomization group
Chang et al., 2007	Cohort	<84	97,275,280	United States	>665 vs. ≤75 mg/d	1.96 (1.11–3.46)	Adjusted for race, total energy intake, parity, oral contraceptive use, strenuous exercise, wine consumption, and menopausal status/hormone therapy use; stratified by age at baseline
Thomson et al., 2008	Cohort	50–79	133,614,451	United States	>130 vs. <58 mg/d	1.07 (0.77–1.48)	Adjusted for age, log calories, No. breast/ovary cancer relatives, dietary modification randomization arm, hysterectomy status, minority race, pack-years smoking, physical activity, nonsteroidal anti-inflammatory drug use, parity, infertility, duration of oral contraceptive use, lifetime ovulatory cycles, partial oophorectomy, age at menopause, and HT usage at entry
Gifkins et al., 2012	PBCC	>21	595,205	United States	>141.8 vs. <82.3 mg/d	1.29 (0.72–2.29)	Adjusted for age (continuous), education, race, age at menarche (continuous), menopausal status and age at menopause for postmenopausal women, parity, OC use, HRT use, BMI (continuous), tubal ligation, and total calories, physical activity (METs), and smoking status
Terry et al., 2017	PBCC	20–79	1,038,406	United States	>142.1 vs. <57.0 mg/d	1.05 (0.66–1.69)	Adjusted for age, region, education, parity, oral contraceptive use, menopause, tubal ligation, family history, BMI, smoking status, total energy, and physical activity

Abbreviations: CI, confidence intervals; HBCC, hospital-based case–control study; PBCC, population-based case–control study; RR, relative risk.

### Dietary vitamin C intake and ovarian cancer risk

**Figure 2:**
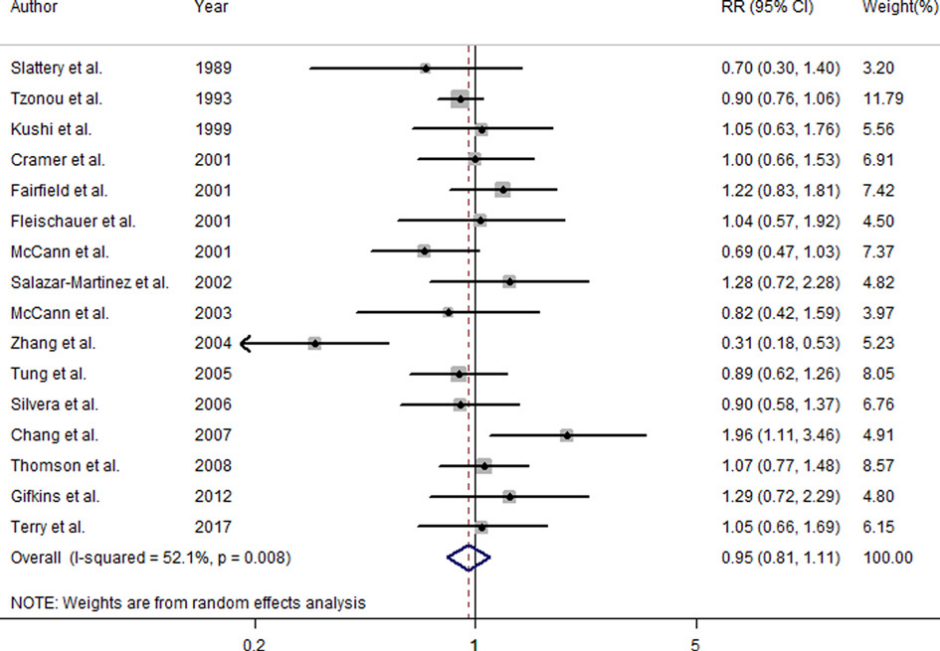
The forest plot between dietary vitamin C intake and ovarian cancer risk

Pooled RR suggested that highest category of dietary vitamin C intake was not associated with the risk of ovarian cancer (RR = 0.95, 95%CI = 0.81–1.11, *I*^2^ = 52.1%, *P*_for heterogeneity_ = 0.008) (Figure 2), when compared with the lowest category. As seen in Figure 2, the association was not significant between dietary vitamin C intake and ovarian cancer risk in North America populations (RR = 1.02, 95%CI = 0.90–1.15, *I*^2^ = 2.0%, *P*_for heterogeneity_ = 0.427). Subgroup analysis by study design got a consistent result both in case-control studies (RR = 0.86, 95%CI = 0.71–1.04, *I*^2^ = 51.8%, *P*_for heterogeneity_ = 0.023) and in cohort studies (RR = 1.15, 95%CI = 0.93–1.42, *I*^2^ = 20.2%, *P*_for heterogeneity_ = 0.286).

### Publication bias and sensitivity analysis

**Figure 3:**
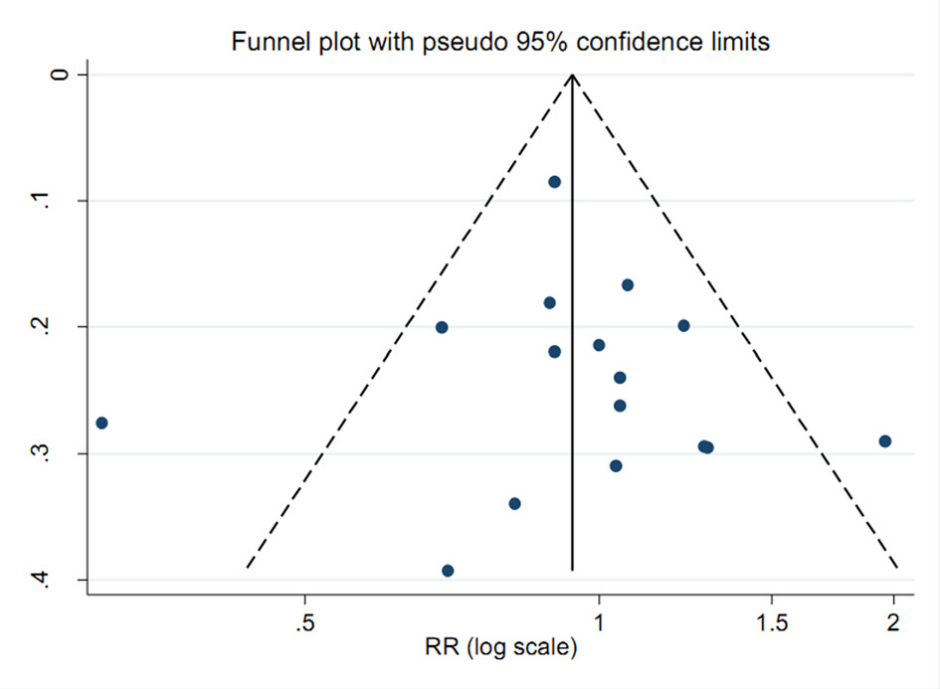
Begg’s funnel plot for publication bias of vitamin C intake and ovarian cancer risk

**Figure 4:**
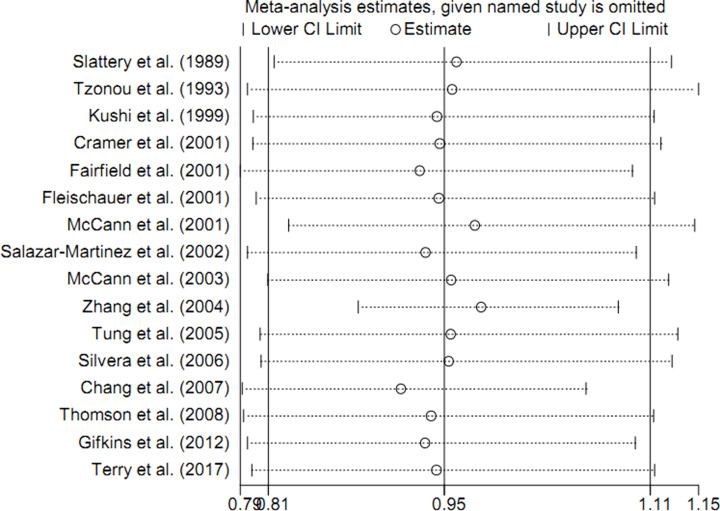
Sensitivity analysis about vitamin C intake on the risk of ovarian cancer

The funnel plots were symmetry on visual inspection (Figure 3). Results of Egger’s regression tests also did not indicate significant publication biases (*P* = 0.790). Sensitivity analysis showed that no single study had a potential influence on the pooled result (Figure 4).

## Discussion

In the current meta-analysis of 16 studies with 4553 cases and 437,689 participants, we found that the highest category compared with the lowest category of dietary vitamin C intake had no significant association on the risk of ovarian cancer. Moreover, by pooling the subgroup results of geographic locations and study design, we got consistent results with the overall result.

Significant heterogeneity (*I*^2^ = 52.1%, *P*_for heterogeneity_ = 0.008) was found in the overall result about vitamin C intake on the risk of ovarian cancer. As far as we know, between-study heterogeneity is common in a meta-analysis, and it is an essential part to explore the sources of heterogeneity. We used meta-regression to explore the causes of heterogeneity for covariates of publication year, study design, geographic locations, and number of cases. We found that geographic locations (*P* = 0.017) may be a covariate that could influence this high heterogeneity. As seen in Figure 2, when we did the hierarchical analysis by geographic locations, the heterogeneity in North America was very low (*I*^2^ = 2.0%, *P*_for heterogeneity_ = 0.427). The *I*^2^ in Europe and Asia was not detected due to only one study in each group. Even though, the result in North American populations was consistent with the overall result.

Although dietary vitamin C intake that is one of antioxidants had some potential role on preventing of cancers [6,8,10] due to inactivating free radicals and reducing oxidative DNA damage, we did not obtain an inverse association between dietary vitamin C intake and ovarian cancer. In our included studies, almost all researches got a non-significant relationship about vitamin C intake on the risk of ovarian cancer. The study by Chang et al. [30] indicated that dietary vitamin C intake (>665 mg/day vs. ≤75 mg/day) could significantly increase the risk of ovarian cancer. The value of highest category (>665 mg/day) was more than that in any other included studies. Otherwise, Zhang et al. [27] suggested that dietary vitamin C intake (≥140.25 mg/day vs. ≤66.50 mg/day) had a lower development on ovarian cancer risk. To our attention, the value of highest category (≥140.25 mg/day) was almost the lowest among all studies. Therefore, the current evidence showed that large amount of dietary vitamin C could not reduce the risk of ovarian cancer, and there may be harm.

Our study has some limitations which should be considered in interpreting the results. First, significant heterogeneity was detected among all the included studies, but it can be successfully explained by a covariate of geographic location. The association was not changed in North America populations. Second, only the subgroup analyses by geographic locations and study design were performed due to the limitation information provided in each individual study. Third, as a meta-analysis of observational studies, although all the included studies were adjusted for age, some related factors such as body mass index (BMI), total energy intake, duration of oral contraception use, and so on were not fully adjusted in every study. Fourth, almost all the included studies come from North America; therefore, more studies conducted in other populations are warranted to further explore the association between geographic locations and ovarian cancer risk. Finally, since we did not get a positive association between dietary vitamin C intake and the risk of ovarian cancer, the dose–response analysis between them was not performed.

## Conclusions

In summary, findings from the present study indicated that dietary vitamin C intake is not associated with the risk of ovarian cancer. Further large-scale cohort studies should be conducted to explore the effect of dietary vitamin C intake on the risk of ovarian cancer due to some limitations existed in our research.

## Abbreviations

CI: confidence interval
RR: relative risk
